# Is there an association between total physical activity level and VO_2max_ among fitness club members? A cross-sectional study

**DOI:** 10.1186/s13102-022-00503-4

**Published:** 2022-06-17

**Authors:** Elene Mauseth Tangen, Christina Gjestvang, Trine Stensrud, Lene A. H. Haakstad

**Affiliations:** Department of Sports Medicine, Norwegian School of Sport Sciences, Ullevål Stadion, P.O Box 4014, 0806 Oslo, Norway

**Keywords:** Fitness club members, Maximal oxygen uptake, Novice exercisers, Physical activity

## Abstract

**Background:**

Since cardiorespiratory fitness is an important predictor for all-cause mortality, it is of interest to know if meeting the physical activity (PA) recommendations is associated with higher levels of maximal oxygen uptake (VO_2max_). We aimed to investigate the association between total PA level given as counts per minute (cpm) and minutes in moderate-to-vigorous PA (MVPA), and VO_2max_ in new fitness club members.

**Methods:**

A total of 62 men and 63 women (≥ 18 years), defined as healthy (no disease considered to hinder PA) participated in this study. VO_2max_ (mL kg^−1^ min^−1^) was measured with a cardiopulmonary exercise (modified Balke protocol), and total PA level was measured with ActiGraph GT1M for seven consecutive days. All participants accumulating ≥ 10 h of activity recordings ≥ 4 days were included in the data analysis. To examine associations between PA level and VO_2max_, a Pearson correlation and a multiple linear regression analysis adjusted for covariates were used.

**Results:**

VO_2max_ (mL kg^−1^ min^−1^) was 40.5 ± 7.2 in men and 35.1 ± 6.0 in women. Total PA level (cpm) and MVPA (min) were 352.4 ± 123.4 and 260.0 ± 132.6 in men and 361.4 ± 103.8 and 273.2 ± 137.0 in women. Total PA level (men: r = 0.346, *p* < 0.01, women: r = 0.267 *p* < 0.01) and MVPA (men: r = 0.359, *p* =  < 0.01, women: r = 0.236, *p* = 0.03) was associated with VO_2max_. When adjusting for age and body fat percentage, total PA level and MVPA were no longer associated with VO_2max_ (men: *p* = 0.11 and *p* = 0.79, women: *p* = 0.40 and *p* = 0.61). In men, age (β = − 0.469 *p* < 0.01) and body fat percentage (β = − 0.483, *p* < 0.01) were the strongest predictor for VO_2max_. For women, body fat percentage was the strongest predictor for VO_2max_ (β = − 0.483, *p* < 0.01).

**Conclusions:**

Total PA level and MVPA were associated with VO_2max_, but the association was low and diminished when adjusted for age and body fat percentage. Body fat percentage (men and women) and age (men) were more strongly associated with VO_2max_ than total PA level and MVPA.

## Introduction

Physical inactivity, defined as not meeting the current recommendations of moderate-to-vigorous physical activity (MVPA) of 150 to 300 min per week, has been identified as the fourth leading risk factor for global mortality [1]. Inactivity is also associated with numerous non-communicable chronic diseases (NCDs) and premature deaths; worldwide, 1.6 million deaths annually related to NCDs can be attributed to low levels of physical activity (PA) [2].

In literature, cardiorespiratory fitness is defined as a measure of total body health and may therefore be an intermediate variable between PA level and health outcomes [3]. Moderate or high levels of cardiorespiratory fitness can prevent numerous NCDs in both sexes regardless of other risk factors [3–5]. It can also predict mortality in adults similarly to traditionally assessed risk factors such as smoking, hypertension, and type 2 diabetes [6, 7].

Cardiorespiratory fitness, usually expressed and measured as maximal oxygen uptake (VO_2max_), is reported in some studies as an indication of PA level [6, 8]. Due to PA’s impact on structural and functional adaptions in the body leading to a greater oxygen transport system, it is observed a graded dose–response change in VO_2max_ by increased PA [3, 6, 9–11]. However, VO_2max_ is largely genetically determined at the individual level and is not shown to be independently influenced by PA pattern only [6, 8, 12–15]. Individual differences in response to PA, as well as current training status, can cause discrepancies concerning this association [7, 12].

While the association between PA and cardiorespiratory fitness is well known in the literature, previous studies have underlined that PA performed in a structural training regimen is essential to obtain an increase in VO_2max_ [8]. Yet, the association between existing PA level and VO_2max_ is still somewhat unclear. For instance, Dyrstad et al. [16] found that relatively large variations in PA level reflected small variations in VO_2max._ A large number of previous publications within this field also rely on self-reported PA in the data analysis, thus potentially causing a higher risk of methodical error [17–19]. Considering individual differences in VO_2max_ in response to PA, this association should be further explored in different populations [20]. This study may fulfill gaps in the literature, as to our knowledge, no former studies have investigated this association utilizing device-measured PA in a population of novice exercisers joining one of the most popular arenas for leisure-time PA, a fitness club [21].

The present study aimed to investigate the association between device-measured PA, (total PA level measured as counts per minute (cpm) and minutes in MVPA), and VO_2max_ in men and women at start-up of fitness club membership.

## Materials and methods

### Design and participants

This cross-sectional study was part of a prospective study conducted in Oslo, Norway, from October 2015 to October 2018, following a group of 125 (62 men and 63 women) newly registered novice exercisers at 25 fitness clubs. In Europe, September and January are two major months for recruiting new fitness club members. Hence, the participants for the present study were mainly enrolled during those two key periods (n = 75, fall, September, and n = 50, winter, January). More details on this study can be found in previous publications [22, 23].

All new members (≤ 4 weeks of a fitness club membership) from 25 multipurpose fitness clubs (a wide range of exercise concepts, resistance and cardio-exercise rooms, group exercise classes, and personal training) were approached to take part in the study by an email invitation from their local gym. Eligibility criteria were: ≥ 18 years of age, reporting exercising ≤ 60 min per week at a moderate or vigorous intensity or brisk walking ≤ 150 min per week in the last six months, healthy (defined as having no chronic disease or pathology considered to hinder PA (i.e. lung disease, heart disease) and not pregnant). A total of 275 new members wanted to participate. Of these, 146 were excluded due to exercising regularly, and 4 due to cardiovascular disease and hypertension.

### Outcome measures

All measures in this study were gathered from baseline measurements done within the first four weeks of a fitness club membership. Information related to demographic and lifestyle variables was obtained via an electronic questionnaire, covering sex, age, smoking, level of education, total household income, and occupation.

#### Maximal oxygen uptake

VO_2max_ (mL kg^−1^ min^−1^) was measured with a cardiopulmonary exercise test (CPET). The CPET was conducted on a treadmill using an incremental modified Balke protocol until exhaustion [24, 25]. VO_2max_ was measured with indirect calorimetry (Oxycon Pro; Jaeger, Hoechberg, Germany). The participants breathed through a Hans Rudolph mask (2700 series, Hans Rudolph, Kansas City, Kansas, USA), which covered the mouth, and was nose-attached to a non-rebreathing tube. The gas exchange variables were continuously sampled and reported as 30 s averages during the CPET. To measure the participants’ maximal heart rate, a heart rate monitor (Polar RS800) was used. The participants started with a 3-min warm-up at an initial speed of 4.5 km/h with no inclination. The inclination increased by 5% every minute up to 20%. The speed was kept constant at 4.5 km/h. When inclination reached 20%, the speed increased by 0.5 km/h every minute, while inclination was kept constant (20%). The Borg Scale (range 6–20) [26] was used for the rating of perceived exertion by participants. To verify a valid VO_2max_, an additional criterion before stopping was a respiratory exchange rate (RER) between 1.10 and 1.30, dependent on age [27]. All measures were calibrated after manufacture guidelines, and the same researcher supervised all CPETs. For accurate measures of VO_2max_, measures of body weight (in kg) and body fat (in percentage) were measured with Inbody 720 (biospace), and height (in cm) was measured with a stadiometer (Seca scale, Mod: 8777021094, S/N: 5877248124885) before the exercise test.

#### Total physical activity level and minutes of moderate-to-vigorous physical activity

Total PA level (in cpm) and MVPA (in min) were assessed with ActiGraph, model GT1M. The participants were all given the same instructions, including how to wear the accelerometer prior to the week of measurement. The accelerometers were worn on the hip for seven consecutive days. All participants who accumulated a minimum of 10 h of activity recordings daily for ≥ 4 days of the seven were included in the data analysis.

The accelerometer measure vertical accelerations in units called counts, and samples data in sampling intervals (epochs). Total cpm is a measure of total PA level and is expressed as the total number of registered counts for all valid days divided by wearing time. Different intensities of PA with count thresholds corresponding to the energy cost of the given intensity were also applied in the data analysis (sedentary behavior: < 100 cpm, light intensity PA: 100–2019 cpm, MVPA: ≥ 2020 cpm). To define proportions meeting PA recommendations (> 150 min of weekly MVPA), the total amount of MVPA during the measurement period was summed up and the number was then divided by the number of days with valid registration [28, 29].

At the time of data analysis for this study, current PA recommendations did not require MVPA to occur in bouts of 10 continuous minutes (with allowance for interruptions of 1–2 min). For this reason, we did not require bouts of 10 min in order to define sufficient and low active participants [30].

### Statistical analysis

Data analysis was conducted using the IBM SPSS Statistics 28.0 program for Windows. The data was first tested for normality using a Kolmogorov–Smirnov test before univariate analysis was performed [31].

In all statistical analyses, the dataset was first grouped by sex due to significant differences in VO_2max_ values [5, 9, 12, 13, 15]. A Pearson correlation was performed for total cpm and VO_2max_, as well as for MVPA and VO_2max_. The correlation values were interpreted as strong (0.50–1.0), moderate (0.30–0.49) and weak (0.10–0.29) [32].

To interpret health factors (BMI, weight, smoking status and body fat percentage) as covariates for this association, univariate analysis was performed on participants above or below references values for VO_2max_ [25]. Body fat percentage, Body Mass Index (BMI) and weight were significantly different between the two groups. Collinearity variables were excluded (BMI and weight). Age was also included as a covariate and found to significantly affect VO_2max_ values, with increasing age corresponding to lower VO_2max_ values in both sexes [18, 25]. The predictive power of these selected covariates (age and body fat percentage), as well as total PA (cpm) and MVPA with VO_2max_, were further analyzed using a multiple linear regression, separately for men and women.

Preliminary analysis was performed to ensure that there were no violations of the assumptions of linear regression, showing normal distribution and absence of multicollinearity as well as absence of outliers in all variables [33]. Coefficient of determination (R^2^) was used to evaluate the precision of the regression, while unstandardized beta (*B*) and standardized beta (β) were used to evaluate each factor’s association with VO_2max_.

Level of significance was set as *p* < 0.05 for all analyzes.

## Results

### Background variables

Table 1 displays the background variables for the participants. Age (years) ranged from 18–71 (mean: 38.8 ± 11.7) in men, and 21–59 (mean: 34.8 ± 10.0) in women (*p* = 0.04). For BMI measures, 57% of the men and 33% of the women were classified as overweight with a BMI of > 25 (*p* < 0.01). A total of 11.5% of the men and 9.5% of the women were classified with obesity (BMI > 30) (*p* = 0.72). More details on this study can be found in previous publications [22, 23].

**Table 1:** Comparison of demographic and socioeconomic variables between men and women

Descriptives	Men *n* = 62	Women *n* = 63	*p* value
Age (yrs)	38.8 ± 11.7	34.8 ± 10.0	0.04
Height (cm)	182.4 ± 7.2	167.4 ± 5.8	< 0.01
Weight (kg)	85.5 ± 12.5	68.8 ± 12.6	< 0.01
BMI (kg/m^2^)	25.6 ± 3.2	24.6 ± 4.5	0.13
Body fat (%)	20.0 ± 3.2	30.5 ± 7.9	< 0.01
Smokes daily	3 (4.8%)	4 (6.3%)	0.71
Higher education > 4 yrs	26 (41.9%)	31 (49.2%)	0.08
Household income > 850,000 NOK	22 (35.5%)	19 (30.2%)	0.53

Data are presented as mean (SD) for continuous variables and n (%) for categorical variables. P-value shows sex differences. BMI, Body Mass Index

The participants completed an average of 6 days with valid PA recordings, with a mean of 13 h wear time daily (Table 2). No sex differences were found in total PA level (cpm) (*p* = 0.65) or MVPA (*p* = 0.58). VO_2max_ was higher among men compared with women (mean diff: 5.4 mL kg^−1^ min^−1^ 95% CI [3.1, 7.8], *p* < 0.01). 24.4% of the men and 39.0% of the women were above reference values for VO_2max_ specific for age and sex [25].

**Table 2:** Comparison of PA level and VO2max (mL kg^−1^ min^−1^) between men and women

	Men *n* = 62	Women *n* = 63	*p* value
**Total physical activity level**			
*Accelerometer recordings*			
Days with valid recording	6.3 ± 1.3	6.4 ± 1.9	0.72
Mean daily wear time (hours)	13.7 ± 1.3	13.85 ± 0.9	0.51
Total PA (cpm)	352.4 ± 123.4	361.4 ± 103.8	0.65
MVPA (min)	260.0 ± 132.6	273.2 ± 137.0	0.58
≥ *150 min of moderate PA/weekly*	44 (66.1%)	50 (79.4%)	0.09
≥ *75 min of vigorous PA/weekly*	7 (11.3%)	7 (11.1%)	0.98
**VO**_**2max**_** assessment**			
*Maximal exercise test using the stepwise Balke protocol*			
VO_2max_ (mL kg^−1^ min^−1^)	40.5 ± 7.2	35.1 ± 6.0	< 0.01
> *Reference values for age group*	15 (24%)	25 (39.7%)	0.06
Time to exhaustion	10.4 ± 1.50	9.0 ± 1.19	< 0.01
Maximal heart rate	182.50 ± 15.1	176.3 ± 25.7	0.10
Borg scale (1–20)	19.3 ± 0.6	19.1 ± 0.7	0.20
RER	1.38 ± 0.1	1.36 ± 0.1	0.21

Data are presented as mean (SD) for continuous variables and n (%) for categorical variables. The p-values shows sex differences. Cpm,counts per minute. MVPA, moderate-to-vigorous physical activity, PA, physical activity, VO_2max_, Maximal oxygen uptake, RER, respiratory exchange ratio

### Device-measured PA associated with VO_2max_

Pearson correlation coefficient (r) revealed a moderate correlation between total PA level and VO_2max_ (r = 0.346, *p* < 0.01) as well as MVPA and VO_2max_ (r = 359, *p* < 0.01) among men (Table 3). In women, we observed a weak correlation between total PA level and VO_2max_ (r = 0.267, *p* = 0.02), as well as MVPA and VO_2max_ (r = 0.236, *p* = 0.03). Age and VO_2max_ showed a strong negative correlation in men (r = − 0.688, *p* < 0.01) and a moderate negative correlation in women (r = − 0.466, *p* < 0.01). Body fat percentage showed a strong negative correlation in both sexes (women: r = − 0.712, *p* < 0.01 and men r = − 0.688, *p* < 0.01).

**Table 3:** Pearsons’ Correlation between total PA level, MVPA, and covariates, and VO_2max_ for both sexes

	VO_2max_ (mL kg^−1^ min^−1^)			
	Men *n* = 62	*p* value	Women *n* = 63	*p* value
Total PA level (cpm)	0.346	< 0.01	0.267	0.02
MVPA (min)	0.359	< 0.01	0.236	0.03
Age (yrs)	− 0.688	< 0.01	− 0.466	< 0.01
Body fat (%)	− 0.650	< 0.01	− 0.712	< 0.01

Cpm, counts per minute, MVPA, moderate-to-vigorous physical activity, PA, physical activity, VO_2max_, maximal oxygen uptake

The adjusted R^2^ of the multiple linear regression was high (R^2^ = 0.682 and 0.577, Table 4), and total PA level, MVPA, age, and body fat percentage in total explained 68% and 57% of the variance in VO_2max_ among men and women, respectively (*p* < 0.01).

**Table 4:** Multiple linear regression summary for factors predicting VO_2max_ mL kg^−1^ min^−**1**^ in both sexes

	B	t	β	*p* value
**Men** *n* = 62 R^2^ = 0.682				
Constant	60.315	19.876		
Total PA level (cpm)	0.014	1.629	0.230	0.11
MVPA (min)	− 0.002	− 0.243	− 0.038	0.79
Body fat (%)	− 0.624	− 6.038	− 0.469	< 0.01
Age (yrs)	− 0.297	− 5.942	− 0.483	< 0.01
**Women** *n* = *63* R^2^ = 0.577				
Constant	51.985	16.361		
Total PA level (cpm)	0.006	0.845	0.104	0.40
MVPA (min)	0.003	0.510	0.063	0.61
Body fat (%)	− 0.471	− 7.084	− 0.618	< 0.01
Age (yrs)	− 0.158	− 2.945	− 0.261	< 0.01

β, Standarized beta, B, Unstandarized beta, Cpm, counts per minute, MVPA, Moderate-to-vigorous physical activity, PA, physical activity, R^2^, adjusted R-square

In both sexes, when adjusting for age and body fat percentage, total PA level and MVPA were no longer significantly associated with VO_2max_ (men: *p* = 0.11 and *p* = 0.79, women: *p* = 0.40 and *p* = 0.61, respectively).

In men, increased age and body fat percentage showed the strongest negative association with VO_2max_ (β = 0.483 and β = − 0.469, *p* < 0.01), whereas for women, increased body fat percentage showed the strongest negative association with VO_2max_ (β = − 0.618, *p* < 0.01).

### Discussion

In this study, we found a moderate association between total PA level and MVPA with VO_2max_ among men. The same results were found in women, yet the association was weaker. When adjusting for age and body fat percentage, we found no association between total PA level or MVPA and VO_2max_ in either of the sexes. Thus, age and body fat percentage may be better predictors than PA level on VO_2max_.

Previous research within this field has observed that PA done at higher intensities (vigorous PA) more strongly influences VO_2max_ [7, 16, 17, 19, 34–36]. The majority of our participants performed moderate PA, and in line with our findings, several studies have not observed an association between moderate PA and VO_2max_ alone [19, 34]. Only 11.3% of the men and 11.1% of the women in our study met the current recommendations of vigorous PA (> 75 min per week) [30]. Using this cutoff, our sample size would have been very small and difficult to derive conclusive evidence from, particularly when doing subgroup analyses assessing the association between device-measured PA and VO_2max_.

When comparing our participants with population studies, 24.0% of the men and 39.7% of the women were above reference values for VO_2max_ [25]. Individuals with higher VO_2max_ values at beginning of a training period have been found to demand an intensity of 85% or higher of maximal heart rate to achieve improvement in VO_2max_ [6]. Ross et al. [7] also stated that most healthy individuals are trainable if training regimes at higher intensities are conducted. Consequently, for this group, we believe that higher intensity PA is needed to affect their VO_2max_.

Intra-individual day-to-day variability of PA may have possibly influenced our findings [37]. PA can vary daily, while cardiorespiratory fitness remains relatively stable, or eventually improves over time with exercise and PA [37]. As such, the measurement period could be biased to reflect the individuals’ PA level, where this study only provided a glimpse of the participant PA level from the week measured. While wearing an accelerometer, the participants may also be more aware of their activity habits (known as reactivity), therefore achieving a higher PA level than under normal circumstances [38]. However, two other studies have concluded that there is not enough evidence supporting reactivity to influence the percentage of the population meeting the current PA recommendations, and MVPA was not influenced by reactivity [38, 39].

Individual differences due to the genetic distribution of VO_2max_ were not accounted for in this study. The majority of our participants met the current PA recommendations when we did not adjust for bouts of 10 min [30]. Additionally, 61.3% of the men and 76% of the women had a VO_2max_ value below reference values [25]. Thus, this indicates that the current PA recommendations may not be sufficient enough to obtain a greater VO_2max_ for all individuals [12, 14, 30]. There are clear biological factors related to oxygen transport or muscular strength that is independent of PA habits, and genetics may be responsible for as much as 50% of the variation in measured VO_2max_ [12, 18, 40]. The adaption in VO_2max_ from PA can therefore vary at any age and in both sexes.

Our study found a stronger correlation between total PA level and MVPA among men compared with women. Some evidence suggests that women experience less adaption than males in response to long-term training, resulting in a smaller increase in VO_2max_ [41]. Thus, the consequence of exercise on VO_2max_ may be generally greater in men than women. However, our study observed no sex differences in PA level as found among Norwegian adults [29]. This indicates that the sex differences found in the present study may be due to morphological and physiological differences between men and women [13, 42]. For instance, we observed that women who participated in this study had a significantly higher body fat percentage compared with men, which in turn may have influenced their VO_2max_ [43].

Increased age and body fat percentage were associated with a decreased level of VO_2max_ in both sexes. Our regression model predicted a decrease of 2.97 and 1.58 (mL kg^−1^ min^−1^) per 10-year of increasing age among men and women, respectively. Inactive individuals VO_2max_ is estimated to decline about 8–10% per decade after the age of 30 [13, 44]. However, due to the research aim of the present study, we did not analyze age differences related to VO_2max_ values.

In line with our results, Mondal and Mishra [45] found a strong negative association between increased body fat percentage and VO_2max_. Around 50% of our participants were classified with overweight (≥ 25 BMI), and 10% with obesity (≥ 30 BMI). This may influence our findings since “The National Health and Nutrition Examination survey” indicates a significantly lower VO_2max_ among individuals categorized with overweight and obesity [46]. Further, Hansen et al. [29] reported that in Norwegian adults, individuals with obesity and overweight had lower odds of meeting the current PA recommendations. This may also indirectly influence one’s VO_2max_. A graded response in lower body fat percentage with increased PA level is also confirmed in both men and women [43].

### Methodological considerations

ActiGraph GT1M cannot accurately identify all forms of PA (e.g. swimming, cycling, upper body movement), account for higher added mass (e.g. carrying a backpack), or isometric muscle contractions (e.g. holding something) [29, 47]. We also used a hip-placed accelerometer, which may also result in limitations to accessing vigorous PA measurements [16, 48]. Brage et al. [48] found that ActiGraph counts peaked when running speed was at 10 km/h, then leveled out when speed was further increased. However, Cleland et al. [49] found that hip placement was the optimal placement to capture a variety of activities, and hip-placed accelerometers are more convenient when measuring free-living PA [50].

Our chosen cut-points, which were applied for cpm may also be a reason why we did not find any strong association between PA and cardiorespiratory fitness. For instance, Miller et al. [51] showed that a higher cpm is needed to define MVPA than what was applied to our participant group. Possibly, no association was found because a large amount of light PA was included as MVPA. As shown, light intensity PA may not be sufficient to improve cardiorespiratory fitness [6, 7]. However, the chosen cut-points for cpm in the present study are both common and widely used in other studies [28, 29, 52].

To the best of our knowledge, this is the first study that investigates the association between PA and VO_2max_ when PA is not adjusted for 10 min bouts of activity according to the new and updated PA recommendation [30]. Due to this, the majority of our participants met the recommendations of 150 min MVPA per/week after the exclusion of bouts of 10 min. We have previously found that 38% of this current participant group met the current PA recommendations when adjusting for 10 min bouts [22]. This shows that the prevalence of sufficient active individuals is substantially higher when bouts are no longer required. The participants were also newly registered fitness club members, and there is a possibility that they had started exercising at their fitness club when they underwent measures of PA level.

### Strengths and limitations

Strong aspects of this study were a sample size (n = 125), with an equal number of men and women, and representing a wide age range. Assessment of VO_2max_ using CPETs is also considered as the most valid measure of cardiorespiratory fitness [53]. The modified Balke protocol was also an appropriate measurement method for our study population (novice exercisers newly registered at a fitness club). We used a device-measurement method (ActiGraph GT1M) to measure total PA level and minutes spent in MVPA. The same researcher tested all participants, reducing the risk of measurement error and improving study results’ reliability.

Study limitations were the use of a uniaxial accelerometer, which may underestimate upper body and horizontal movements (such as cycling and resistance training). Bahls et al. [8] reported that cardiorespiratory fitness was not associated with all forms of PA (for instance work-related PA) and was greater influenced by structural PA. However, we did not control for which type of PA the participants conducted. The measurement period of PA may also not represent the participants’ PA habits, and we speculate that the measurement period may have either overestimated the participants’ PA levels or did not represented overall PA levels due to day-to-day variability. It is also widely known that p-values are dependent on sample size, which may be the reason for the lack of a strong statistically significant association between PA and VO_2max_ in this study [54]. Thus, we do not know if this non-significant finding would still be present if we had recruited more participants, and thus achieved a higher statistical power.

Considering that the present study had a generally low response rate, there is uncertainty about whether the representativeness of our sample represents the target population (novice exercisers at fitness clubs). Data were only obtained from a multipurpose fitness club chain in an urban area of Norway. Recruitment from other gym segments (of low to high membership fees) could have provided different results. Thus, the study cannot exclude the risk of selection bias. However, the chosen fitness club segment is a large chain and including other fitness clubs would likely have increased the heterogeneity of the participant groups. The results would therefore be more difficult to interpret.

## Conclusion

We found an association between both device-measured total PA level and MVPA and VO_2max_ in healthy men and women at the start-up of a fitness club membership, but the association was low and further diminished when adjusting for age and body fat percentage. Body fat percentage (men and women) and age (men) were found to be more strongly associated with VO_2max_ than total PA level and MVPA in both sexes.

## Data Availability

The datasets generated and analysed in this study are not publicly available since it were used under license for the current study, and so are not publicly available. Data are available from the corresponding author on reasonable request.
